# Non-coding somatic single-nucleotide variations affecting glioblastoma-specific enhancer elements regulate tumor-promoting gene networks

**DOI:** 10.1016/j.gendis.2025.101762

**Published:** 2025-07-05

**Authors:** Sandra Iñiguez-Muñoz, Pere Llinàs-Arias, Miquel Ensenyat-Mendez, Andrés F. Bedoya-López, Maria Solivellas-Pieras, Santiago Garfias-Arjona, Mónica Lara-Almúnia, Gabriel Matheu, Ananya Roy, Karin Forsberg-Nilsson, Diego M. Marzese

**Affiliations:** a Cancer Epigenetics Laboratory at the Cancer Cell Biology Group, Institut d'Investigació Sanitària Illes Balears (IdISBa), Palma 07120, Spain; b Department of Neurosurgery, Hospital Quirónsalud Palmaplanas, Palma 07011, Spain; c Department of Neurosurgery, Jimenez Diaz Foundation University Hospital, Madrid 28040, Spain; d Ruber International Hospital, Madrid 28034, Spain; e Department of Pathology, Son Espases University Hospital (HUSE), Palma 07210, Spain; f Department of Immunology, Genetics and Pathology, Uppsala University, Uppsala 751 85, Sweden; g Science for Life Laboratory, Uppsala University, Uppsala 751 85, Sweden; h University of Nottingham Biodiscovery Institute, Nottingham NG7 2RD, UK; i Department of Surgery, Duke University School of Medicine, Durham, NC 27705, USA; j Duke Cancer Institute, Duke University, Durham, NC 27705, USA

Glioblastoma (GBM) and low-grade glioma (LGG) are brain tumors with distinct molecular features. GBM represents the most aggressive primary brain tumor (median survival = 15 months, 5-year overall survival rate <10%) whose heterogeneity is reflected in distinct gene expression profiles.^1^ Non-coding alterations compromise nearly 98% of the genome and remain largely underexplored.^2^ In this scenario, the Pan-Cancer Analysis of Whole Genomes project detected numerous somatic single-nucleotide variations (SNVs) in gene regulatory elements, suggesting a functional impact on gene regulation. These mutations disrupt transcription factor binding sites, leading to dysregulated transcriptional programs.^3^ Enhancer elements (EEs), a class of gene regulatory elements, regulate proximal and distal gene expression and can become aberrantly activated in cancer, contributing to tumor progression.^3^ This study identified GBM-specific EEs affected by non-coding somatic SNVs in two independent and demographically diverse GBM patient cohorts (Spain and Sweden). Computational modeling demonstrated that these SNVs disrupted transcription factor (TF) binding motifs, altering the affinity of key TFs, such as transcriptional enhancer-associated domain (TEAD), early 2 factor 1 (E2F1), and signal transducer and activator of transcription 3 (STAT3), which are known to drive GBM malignancy. Our findings emphasize the role of non-coding SNVs in reprogramming the GBM epigenetic landscape and driving tumor progression through aberrant TF activity.

To identify dynamically active gene regulatory elements in GBM, we compared chromatin accessibility between GBM (*n* = 9) and LGG (*n* = 12) with assay for transposase-accessible chromatin using sequencing (ATAC-seq) from The Cancer Genome Atlas (TCGA; Table S1).^4^ We identified 22,346 LGG-specific accessible regions and 18,808 GBM-specific accessible regions (Fig. 1A). In LGG, active gene regulatory elements were mostly promoters (*n* = 8475), followed by IEs (*n* = 2279) and EEs (*n* = 777) (Fig. S1A, B). In contrast, GBM-specific regions included 7089 promoters, 2340 IEs, and 1838 EEs (Fig. S1A, C). While promoters were similarly distributed in both tumor types, GBM exhibited a significant enrichment of IEs and EEs (*p* < 0.001; Fig. 1B), with dynamic EEs showing the most pronounced differences (*p* < 0.001; Fig. S1D). Thus, aberrant activation of EEs may play a pivotal role in shaping the transcriptional landscape of GBM, potentially contributing to its aggressiveness.

**Figure 1:**
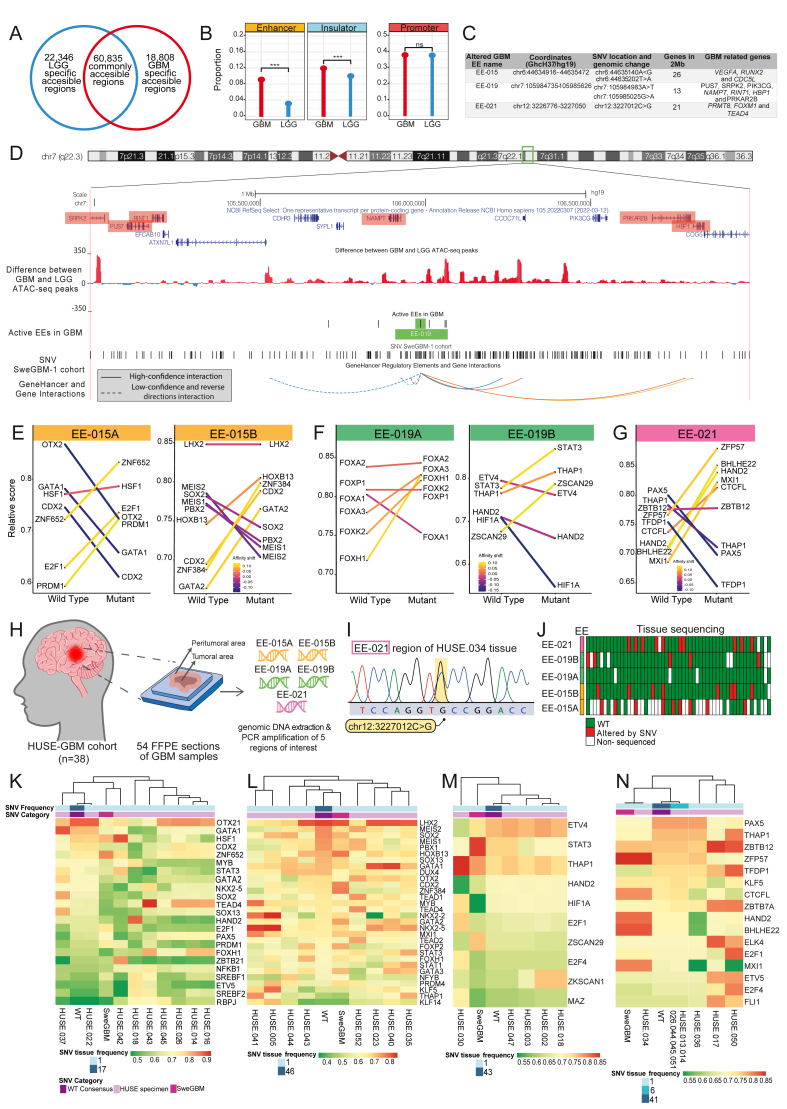
Characterization of GBM-specific active enhancers with non-coding mutations and their role in transcription factor binding dynamics. **(A)** The Venn diagram representing the number of chromatin accessibility GBM-specific active regions (red), LGG-specific active regions (blue), and commonly accessible chromatin segments. **(B)** The lollipop chart displaying the proportion of EEs, IEs, and promoters in GBM-specific active regions and LGG-specific active regions. Chi-square test, ∗∗∗*p* < 0.001; ns for *p* > 0.05. **(C)** GBM-specific dynamic EE candidates with their location, the SNV position, the number of genes in the 2 Mb window, and GBM-related genes in the bibliography. **(D)** UCSC Genome Browser (GRCh37/hg19) 2 Mb view around one of the dynamically altered candidates EEs (EE-019 in green), including the difference between GBM and LGG ATAC-seq chromatin accessibility peaks. GBM-related genes around 2 Mb of the EE are represented in red. The GeneHancer track indicated EE candidate–gene interactions. High–confidence interactions are represented by solid lines, and low–confidence interactions are shown with dashed lines; reverse–direction interactions are indicated by dashed lines. **(E**–**G)** TF binding affinity shifts of the top five TF ranking predictions between wild-type and mutated sequences in dynamic EE candidates. The parallel coordinate plots represent the relative score changes between wild-type and mutant sequences of the top five ranking predicted TFs. We considered a 15 bp sequence with the alteration positioned at the center. The top five ranking predicted TFs: (E) EE-015A chr6:44635140A>G (left) and EE-015B chr6:44635202T>A (right); (F) EE-019A chr7:105984983A>T (left) and EE-019B chr7:105985025G>A (right); and (G) chr12:3227012C>G of EE-021. **(H)** Scheme of SNV detection process on altered EEs in the GBM samples from the HUSE-GBM cohort. **(I)** The simplified heatmap displaying the sequencing status (WT, altered by SNV, or non-sequenced) of all tissues from the HUSE-GBM cohort in each non-coding region of interest. **(J)** Electropherogram of the chr12:3227012C>G SNV detection in the GBM-034 tissue of the HUSE-GBM cohort. **(K–N)** Predicted TF binding pattern with altered motif by SNVs in GBM tissues from the HUSE-GBM cohort located in the candidate EE region. In all heatmaps, we represented SNV frequency and category and the TF binding relative score of altered sequences around (K) chr6:44635140A>G (EE-015); (L) chr6:44635202T>A (EE-015); (M) chr7:105985025G>A (EE-019); and (N) chr12:3227012C>G (EE-021). GBM, glioblastoma; LGG, low-grade glioma; IE, insulator element; EE, enhancer element; TF, transcription factor; SNV, single-nucleotide variation.

Next, we investigated non-coding somatic SNVs in EEs using whole-genome-sequencing data from specimens in the Sweden GBM cohort (SweGBM-1, *n* = 39; Table S2).^5^ Among the 1367 GBM-specific dynamic EEs, we identified 30 harboring somatic SNVs in at least one patient (Table S3). We prioritized three dynamic EEs (EE-015, EE-019, and EE-021) based on the number and recurrence of these non-coding SNVs. Additionally, we considered the functional relevance of GBM-associated genes located within genes located within ±1 Mb, taking also into account predicted EE-genes interactions (Fig. 1D; Fig. S2A, B). In EE-015, two SNVs (chr6:44635140A>G and chr6:44635202T>A) were identified in distinct regions (EE-015A and EE-015B), located near vascular endothelial growth factor A (*VEGFA*), Runt-related transcription factor 2 (*RUNX2*), and cell division cycle 5-like (*CDC5L*). Interestingly, this EE is predicted to bind to the *RUNX2* promoter, being able to modulate its expression (Fig. S2A). Similarly, two SNVs (chr7:105984983A>T and chr7:105985025G>A) were detected within EE-019 (EE-019A and EE-019B), a gene regulatory element which potentially interacts with the GBM-related genes nicotinamide phosphoribosyltransferase (*NAMPT*) and high mobility group box protein 1 (*HBP1*) (Fig. 1D). Additionally, a recurrent mutation (chr12:3227012C>G) found in three patients affected EE-021, a regulatory element located close to relevant genes for this disease, such as protein arginine methyltransferase 8 (*PRMT8*), forkhead box protein M1 (*FOXM1*), and *TEAD4* (Fig. S2B). Collectively, these findings suggest that non-coding somatic SNVs within GBM-specific EEs might influence gene regulatory networks involved in GBM malignancy and treatment resistance.

Then, we analyzed TF binding affinity changes within ±7 bp of each mutation site. In EE-015A (chr6:44635140A>G), the mutation increased the predicted binding affinity for heat-shock transcription factor 1 (HSF1) and introduced a novel binding site for E2F1, both linked to GBM progression (Fig. 1E; Fig. S3A). The second SNV in EE-015 (EE-015B; chr6:44635202T>A) strengthened the predicted binding of homeobox 13 (HOXB13), caudal type homeobox 2 (CDX2), zinc finger protein 384 (ZNF384), and GATA binding protein 2 (GATA2) (Fig. 1E; Fig. S3A). Of note, ZNF384, with increased affinity for EE-015B mutation, has been implicated in glioma progression and temozolomide resistance through microsomal glutathione S-transferase 1 (MGST1) regulation and ferroptosis inhibition, as well as in IFI30-mediated malignant progression. Conversely, this mutation significantly decreased the binding affinity for meis homeobox 1 (MEIS1), MEIS2, PBX homeobox 2 (PBX2), and sex determining region Y-box 2 (SOX2), TFs critical for neural differentiation. In EE-019, we found two distinct SNVs. While the first mutation (EE-019A, chr7:105984983A>T) slightly reduced the affinity of forkhead box protein A1 (FOXA1), increased the affinity of FOXA3, FOXK2, and FOXH1 (Fig. 1F; Fig. S3B, left), the second mutation (EE-019B, chr7:105985025G>A) increased the affinity of STAT3 and Thanatos-associated protein domain-containing 1 (THAP1), TFs involved in GBM proliferation and immune evasion. Besides, heart and neural crest derivatives expressed 2 (HAND2), hypoxia inducible factor 1 subunit alpha (HIF1A), and E26 transformation-specific variant 4 (ETV4) lose their binding affinity with the mutated sequence (Fig. 1F; Fig. S3B). Finally, the SNV affecting EE-021 (chr12:3227012C>G) substantially increased the binding affinity of zinc finger protein 57 (ZFP57) and CCCTC-binding factor-like (CTCFL), both related to glioma progression and epigenetic regulation (Fig. 1G; Fig. S3C). These findings indicate that non-coding SNVs in GBM-specific EEs can reprogram TF binding dynamics, potentially contributing to tumor progression and therapy resistance.

Using targeted sequencing, we validated these GBM-specific SNVs in an independent cohort of 54 formalin-fixed paraffin-embedded GBM tissues from the HUSE-GBM cohort (Spain; Table S4). We focused on SNVs affecting TF binding (EE-015A-B, EE-019A-B, and EE-021; Fig. 1H–J; Fig. S4), generating 230 sequences of these regulatory regions (Table S5). Four out of five regions provided high-quality sequencing data in over 85% of the samples, with EE-015A showing the lowest quality coverage (50%; Fig. 1J; Fig. S4A). EE-015A exhibited the highest mutation frequency (30%), while other regions showed mutations in 10%–20% of samples, except EE-019A, which lacked detectable alterations (Fig. S4A, B). Overall, more than 46% of the samples displayed at least one mutation in these regions, supporting the SweGBM-1 findings. Notably, seven out of 54 samples contained mutations in two distinct regulatory elements, suggesting potential cooperative effects among these non-coding SNVs in modulating GBM gene networks (Fig. S4C). Furthermore, the same EE-021 mutation identified in the SweGBM-1 cohort was also detected in the HUSE-GBM cohort, reinforcing its potential relevance in GBM pathogenesis (Fig. 1I). The remaining SNVs detected in HUSE-GBM were located within 7 bp of the positions initially discovered in SweGBM-1, suggesting a common mutational hotspot and reinforcing the idea that these non-coding SNVs may disrupt TF binding and other epigenetic mechanisms, ultimately driving tumor progression. Interestingly, none of the mutations identified in the HUSE-GBM cohort were known SNPs, suggesting they are somatic in origin and potentially tumor-specific. To assess intra-tumoral heterogeneity, we analyzed SNV profiles across multiple tumor sections from 11 HUSE-GBM patients with multifocal disease. Four of the five analyzed regions showed over 75% concordance between different tissue sections, indicating a consistent mutation profile stable (Fig. S4D). Additionally, sequencing nine peritumoral regions from patients harboring SNVs in their tumor cores revealed no mutations in surrounding non-tumoral tissue (Fig. S5). This exclusivity of SNVs to tumor regions further supports their potential as molecular drivers of GBM progression.

Finally, we investigated how these mutations impact TF binding affinity in GBM tissues from the HUSE-GBM cohort. Mutations in EE-015A, including one detected in HUSE.042, increased HSF1 and *TEAD4* binding, mirroring findings from the SweGBM-1 cohort (Fig. 1K). Mutations in EE-015B enhanced GATA1 and NK2 homeobox 2 (NKX2-2) affinity, both genes related to tumor proliferation and oncogenic signaling (Fig. 1L). Conversely, EE-019B mutations had minimal impact, except in HUSE.030, where increased THAP1 and reduced HAND2 binding were observed (Fig. 1M). Importantly, the EE-021 SNV in HUSE.034 was identical to SweGBM-1. Furthermore, specific mutations in HUSE.017 and HUSE.050 increased the predicted binding of ZFP57 and zinc finger and BTB domain-containing 12 (ZBTB12), two TFs linked to chromatin remodeling and transcriptional repression in gliomas (Fig. 1N).

Our study identifies non-coding somatic SNVs within GBM-specific EEs in two independent cohorts. These mutations modulate the predicted binding of TFs, which may eventually trigger an imbalance in gene expression. Cross-validation in an independent cohort reinforces the relevance of the identified EEs, highlighting their potential regulatory role. These findings provide novel insights into non-coding mutations as drivers of GBM and open avenues for further functional and therapeutic exploration.

## CRediT authorship contribution statement

**Sandra Iñiguez-Muñoz:** Visualization, Formal analysis, Writing – review & editing, Methodology, Data curation, Writing – original draft, Investigation. **Pere Llinàs-Arias:** Writing – review & editing, Investigation, Writing – original draft, Methodology. **Miquel Ensenyat-Mendez:** Methodology, Writing – review & editing, Formal analysis, Writing – original draft, Data curation. **Andrés F. Bedoya-López:** Methodology, Data curation, Writing – review & editing, Investigation, Software, Formal analysis. **Maria Solivellas-Pieras:** Formal analysis, Writing – review & editing, Visualization. **Santiago Garfias-Arjona:** Writing – original draft, Conceptualization, Supervision, Writing – review & editing, Resources. **Mónica Lara-Almúnia:** Writing – review & editing, Methodology, Conceptualization. **Gabriel Matheu:** Supervision, Methodology, Writing – review & editing, Conceptualization. **Ananya Roy:** Writing – review & editing, Resources, Writing – original draft, Conceptualization, Supervision. **Karin Forsberg-Nilsson:** Writing – review & editing, Resources, Writing – original draft, Investigation, Supervision, Conceptualization. **Diego M. Marzese:** Supervision, Writing – review & editing, Funding acquisition, Writing – original draft, Conceptualization.

## Funding

This study was supported by the Instituto de la Salud Carlos III Miguel Servet II (No. CPII22/00004 to D.M.M.), the AES 2019 (No. PI19/01514 to D.M.M.), the AES2022 (No. P22/01496 to D.M.M.), the Sara Borrell Project (No. CD22/00026), the Institut d’Investigació Sanitària Illes Balears (IdISBa) INSE/Marzese and Liberi Program, the Scientific Foundation of the Spanish Association Against Cancer (A.F.B.-L), and the Balearic Islands Government FPI Program (No. FPI/037/2021 to S.Í.-M).

## Conflict of interests

The authors have no competing financial interests to disclose.
