# Supplementary material for: Non-coding somatic single-nucleotide variations affecting glioblastoma-specific enhancer elements regulate tumor-promoting gene networks
Source: Genes Dis. 2025 Jul 5;13(1):101762. doi: 10.1016/j.gendis.2025.101762 (PMC12466127; doi:10.1016/j.gendis.2025.101762)
Supplement: Multimedia component 1 [file mmc1.docx]

**SUPPLEMENTARY DATA**

**Title:** Non-coding somatic single nucleotide variations affecting glioblastoma-specific enhancer elements regulate tumor-promoting gene networks.

**Authors:** Sandra Iñiguez-Muñoz^1^, Pere Llinàs-Arias^1^, Miquel Ensenyat-Mendez^1,9^, Andrés F. Bedoya-López^1^, Maria Solivellas-Pieras^1^, Santiago Garfias-Arjona^2^, Mónica Lara-Almúnia^3,4^, Gabriel Matheu^5^, Ananya Roy^6,7^, Karin Forsberg-Nilsson^6,7,8^, Diego M. Marzese^1,9,10*^.

**Affiliations**

1. Cancer Epigenetics Laboratory at the Cancer Cell Biology Group, Institut d’Investigació Sanitària Illes Balears (IdISBa), Palma, Spain
2. Department of Neurosurgery, Hospital Quirónsalud Palmaplanas, Palma, Spain
3. Department of Neurosurgery, Jimenez Diaz Foundation University Hospital, Reyes Católicos Av., No 2, 28040 Madrid, Spain
4. Ruber International Hospital, Maso St., No 38, 28034 Madrid, Spain.
5. Department of Pathology, Son Espases University Hospital (HUSE), Palma, Spain.
6. Department of Immunology, Genetics and Pathology Uppsala University, 751 85 Uppsala, Sweden.
7. Science for Life Laboratory, Uppsala University, 751 85 Uppsala, Sweden
8. University of Nottingham Biodiscovery Institute, Nottingham, UK.
9. Department of Surgery, Duke University School of Medicine, Durham, NC, USA
10. Duke Cancer Institute, Duke University, Durham, NC, USA

***Corresponding Author Information:**

Diego M. Marzese, PhD

Email: dmarzese@gmail.com

Carretera Valldemossa 79

Palma, Balearic Islands, 07120 Spain.

**SUPPLEMENTARY MATERIALS AND METHODS**

**Data collection**

Chromatin accessibility maps for GBM and LGG were generated by merging the ATAC-seq peak calls from the Cancer Genome Atlas (TCGA Table.S1)^1^. Raw ATAC-seq peak pileup data were downloaded in April 2021 from TCGA and were filtered to exclude peaks that map to chromosome Y or located in non-canonic chromosomes. For this study, LGG (n=12) and GBM (n=9) TCGA patients with available ATAC-seq peak calls were included. To ensure consistency in genomic annotations, hgLiftOver (UCSC) was used to convert ATAC-seq data from human genome assembly GRCh38 to GRCh37^2^. Regions with ATAC-seq peaks detected exclusively in LGG but absent in GBM were classified as LGG-specific accessible regions, while those present only in GBM were designated as GBM-specific active regions. Whole-genome sequencing (WGS) data from the SweGBM-1 cohort^3^ (Table.S2) were analyzed to identify non-coding mutations in GBM tumors. This cohort consists of 39 IDH1 wild-type (IDH1wt) GBM patients, including 31 males and 8 females, with ages ranging from 38 to 83 years (median: 65 years).

**Characterization of gene regulatory elements and their potential regulatory relationships**

Promoter regions were delimited as +/−1,500 bp around the nearest transcription start site (TSS) using NCBI RefSeq (Homo sapiens Feb. 2009 grcH37/hg19), downloaded from the Genome Browser annotation track database. EE annotations were downloaded from the FANTOM5 database^4^. All CTCF motifs from the genome-wide position weight matrix scanner (PWMScan) from the JASPAR CORE 2020 vertebrate motif library^5^ were classified as IE. The *annotatePeak* function from the ChIPseeker R package v3.17^6^ retrieved the annotation of the genomic region information (Promoter ≤1kb, promoter 1-2kb, promoter 2-3kb, 5’ UTR, 3’ UTR, Exon, Intron, Downstream, Intergenic). Consequently, the distribution of active genomic regions was analyzed, considering the following sections: i) Distal intergenic; ii) Intragenic (Exon, Intron, 5’ UTR, 3’ UTR, Downstream); iii) Promoter (Promoter ≤1kb, Promoter 1-2kb, Promoter 2-3kb); iv) IEs; v) EEs; vi) Dual elements (IE-EE regions). To assess the presence of previously reported variants near EE candidates, dbSNP 155 (NCBI) was used^7^. BEDfiles were processed using Bedtools v2.31.0^8^ on the Galaxy open-source platform^9^.

Additionally, to explore potential regulatory interactions between candidate EEs and nearby genes, we incorporated GeneHancer (version v2, available in the UCSC Genome Browser), a database that integrates enhancer-gene associations based on multiple sources, including eQTLs, chromatin interactions, and EE activity data^10^. The GeneHancer track was used to visualize and assess whether our candidate EEs have been previously linked to the regulation of proximal genes.

**Analysis of transcription factor binding sites**

TF binding affinity was evaluated using the 2020 (8^th^ release) JASPAR database^5^. For each analysis, the 15bp sequence containing the SNV (±7 bp window) was used as input to scan for potential TF binding sites. The binding affinity was assessed using selected position weight matrix (PWM) models, applying a 70% relative profile score threshold. The selection criteria in JASPAR were set as follows: CORE Collection, Homo sapiens species, All classes, JASPAR 2020 version, Vertebrates taxonomy, ChIP-seq data type (233 TF matrix profiles), and All families. The top five TF were ranked according to their relative binding score.

**HUSE-GBM validation cohort and processing GBM tissue samples**

For validation, 54 paraffin‐embedded GBM tissue samples from 38 patients were obtained from the Pathology Department of Son Espases University Hospital (**Table.S4**). The cohort included 48.65% male and 51.35% female patients, with ages between 24 to 84 years (median 58 years). Among the patients, 73.34% had unmethylated *MGMT* promoter and 94.6% were *IDH1* wild type. Almost 89.2% of GBM patients received radiotherapy and chemotherapy. All tissue specimens were formalin-fixed, paraffin-embedded, and sectioned at 10 μm in thickness. Clinical data were collected from Son Espases Hospital records. Sample collection for this study was approved by the Ethics Research Committee of the Balearic Islands (Approval No. IB 3161/16 PI) and informed consent was obtained from all subjects.

**DNA isolation from paraffin tissue sections**

Homogenous tumor sections were microdissected using a sterile needle to ensure selective extraction of genomic DNA while minimizing contamination from peritumoral tissue. DNA was isolated using the Quick-DNA FFPE Kit (ZYMO Research Cat.No:D3067) according to the manufacturer´s instructions. DNA concentrations and purity were assessed by spectrophotometric absorbance measurement at 260 nm and 280 nm using the BioTek Synergy H1 Plate Reader (Agilent).

**PCR amplification and Sanger sequencing**

Polymerase chain reaction (PCR) conditions were optimized for the amplification of EEs DNA sequences flanking SNV candidates using HiFi Platinum^TM^ DNA Polymerase (ThermoFisher Scientific, Cat.No: 11304011) following the manufacturer's recommendations. The TProfessional Biometra PCR thermocycler machine was used, and the primer sets are listed in **Table.S6**. PCR was performed using between 50 ng and 100 ng of each DNA sample. Subsequently, PCR products were gel purified with NucleoSpin Gel and PCR Clean-up kit (MACHEREY-NAGEL, Germany). Finally, the purified gel products obtained were sent to Macrogen (Spain) for sequencing by Sanger´s method using sequencing primers (**Table.S7**).

**Data Processing and visualization**

Data processing and visualization were conducted using various R packages. Specifically, *ggplot2* v3.4.0^11^ was used for generating bar plots, providing a clear and precise representation of the data. The *UpSetR* v1.4.0^12^ package was used to illustrate the distribution of active genomics regions, while *pheatmap* v1.0.12 facilitated heatmap generation for visualizing complex data patterns and relationships. The *RColorBrewer* v1.1.3 package was applied to enhance color schemes for improved interpretability. For data transformation, the *tidyverse* v2.0.0 package was used, while the *patchwork* v1.2.0 package enabled the seamless integration of multiple plots into single figures. To visualize TF binding affinity shifts between wild-type and mutant sequences, parallel coordinate plots were generated using *viridis* v.0.6.5 and *gggrepel* v0.9.5.

**Statistics**

Chi-squared tests were performed in RStudio to assess associations between categorical variables. This approach was used to compare the proportions of EEs, IEs, and promoters in GBM-specific and LGG-specific active regions.

**Data availability**

ATAC-seq data used in this study from GBM and LGG projects of TCGA are publicly available as described in the Method section. We utilized previously published WGS data from the SweGBM-1 cohort^3^. Furthermore, we utilized publicly available data of promoter regions defined by NCBI RefSeq (GRCh37/hg19), enhancer annotations from FANTOM5^4^, and CTCF motifs from JASPAR CORE 2020^5^.

**EXTENDED DISCUSSION**

Over recent years, most of the cancer-related discoveries have centered on mutations within coding regions, particularly those involving driver genes^13^. Thus, the role of non-coding somatic mutations in glioblastoma (GBM) pathogenesis has been relatively overlooked. However, emerging studies have increasingly highlighted the significance of mutations beyond coding regions in tumorigenesis^14–18^. Showing that non-coding mutations that alter gene regulatory elements (GREs), such as enhancer elements (EEs), play a pivotal role in modulating tumor progression by controlling the expression of both proximal and distant gene networks^19^. A notable example of non-coding somatic SNVs in GBM is the alteration of the *TERT* promoter mutation^20^, which leads to *TERT* up-regulation and consequently increased telomerase activity^21^. This mutation, reported in multiple malignancies including GBM, creates a new binding site for GABP, a transcription factor that mediates *TERT* overexpression^22^. Our study addresses this gap by identifying non-coding somatic SNVs within GREs specific to GBM. By exploring whole-genome sequencing (WGS) data from a clinically well-annotated GBM discovery cohort (SweGBM-1)^3^ and validating these results in GBM specimens from a validation cohort of patients (HUSE-GBM), our findings reveal novel insights into how these mutations may influence the binding affinity of transcription factors (TFs) and consequently affect gene expression linked to GBM progression. While our study focuses on GBM, these GREs could be relevant in other aggressive tumors.

Specifically, we identified 30 GBM-specific EEs affected by non-coding SNVs in the SweGBM-1 cohort, prioritizing those with higher mutation frequency and proximity to GBM-associated genes. These mutations modulated TF binding affinity, particularly in key oncogenic TFs such as HSF1, E2F1, and STAT3, which are known drivers of GBM tumorigenesis. For instance, mutations in EE-015A created predicted binding sites for HSF1 and E2F1, while mutations in EE-019B enhanced STAT3 binding affinity. These changes can potentially lead to the dysregulation of critical genes involved in GBM progression. Future research should explore the allele-specific effects of these SNVs to determine whether their functional consequences vary depending on the genetic context, which could further elucidate their roles in GBM pathogenesis.

To assess the robustness and clinical relevance of these findings, we validated them in an independent GBM cohort from Spain (HUSE-GBM). This cross-validation across demographically distinct populations is crucial to determine whether the identified mutations are population-specific or represent universal features of GBM. The presence of these mutations in two independent cohorts strengthens the reliability of our findings and lays the foundation for functional studies aimed at elucidating their biological impact. These results suggest that non-coding somatic SNVs within GBM-specific EEs are not just passenger mutations but could actively reshape the gene regulatory landscape in GBM, offering potential therapeutic targets.

Thus, our study revealed several important findings. First, we identified EEs harboring non-coding SNVs associated with GBM progression. Notably, the identified SNVs were located in regions near genes (±1Mb from the interrogated EEs) previously implicated in GBM tumorigenesis, such as *VEGFA*^23^, *RUNX2*^24^, *CDC5L*^25^, and *PIK3CG*^26^, among others. By evaluating TF binding affinity changes induced by these mutations, we identified several TFs whose binding was altered by the presence of SNVs, including well-known GBM-associated TFs such as HSF1^27,28^, E2F1^29–31^, HOXB13^32^, CDX2^33,34^, GATA2^35,36^, FOXP1^37,38^, FOXA2^39^, STAT3^37,40^, ZNF384^41,42^ and TEAD4^43^. Additionally, we identified TFs not previously associated with GBM, such as ZNF652, FOXA3, FOXH1, THAP1, ZSCAN29, BHLHE22, and HAND2. The predicted binding affinity of these TFs suggests that non-coding somatic SNVs can recruit oncogenic TFs, leading to enhanced transcription of tumor-promoting genes. Conversely, these mutations could also disrupt the binding of tumor-suppressive TFs, further contributing to cancer progression.

Our study also highlights the importance of considering the broader regulatory networks in which these GBM-specific EEs may operate. Specifically, the detected EEs are likely part of regulatory networks involving well-established oncogenic and tumor suppressor pathways in GBM, such as the RTK/RAS/PI3K, p53, and cell cycle regulatory networks. The presence of SNVs in EEs can potentially affect the expression of multiple genes within the regulatory landscape, contributing to the complexity of GBM tumorigenesis. By integrating these findings with the existing knowledge of GREs and TF networks, our study improves the understanding of the regulatory mechanisms driving GBM progression. Furthermore, identifying the specific TFs and GREs associated with these EEs helps to map out the regulatory circuits that could be disrupted in GBM due to these non-coding mutations. Given the invasive nature of GBM and the propensity for tumor rim cells to evade treatment, further analysis of the peritumoral area, including cellular and molecular profiling, would be critical to understanding these dynamics and their implications for recurrence.

In summary, our work provides a novel perspective on the regulatory mechanisms underlying GBM by emphasizing the role of non-coding somatic SNVs in EEs. These findings open new avenues for understanding GBM pathogenesis and identifying potential diagnostic or therapeutic targets. Future studies should focus on large-scale cohort analyses and functional assays consisting of ongoing advancements in CRISPR/Cas and base editing technologies to further elucidate the downstream effects of these non-coding mutations and their clinical implications.

**EXTENDED CONCLUSIONS**

This study suggests a critical role of non-coding somatic SNVs in GBM-specific EEs and their potential impact on transcription factor binding and gene expression. By identifying novel mutations in these EEs and validating their presence in independent cohorts, we highlight the significance of non-coding regions in GBM progression. Validating our findings from a cohort of GBM patients from Sweden in an independent cohort from Spain enhances the robustness of our findings, ensuring their applicability across different populations and genetic backgrounds. Our findings pave the way for future research to explore the functional consequences of these mutations and their potential as diagnostic or therapeutic targets. Continued investigation into the non-coding genome is essential for advancing our understanding of the genetic basis of GBM and improving patient outcomes.

**SUPPLEMENTARY FIGURE LEGENDS**

**Supplementary Figure 1. GREs distribution across LGG- and GBM-specific accessible regions using TCGA ATAC-seq data. (A)** Workflow indicating the number of identified GREs in LGG- and GBM-specific active regions differentiating two types of EEs, dynamic and constitutive. **(B)** UpSet plot of the distribution of LGG-specific active regions. **(C)** UpSet plot of the distribution of GBM-exclusive active genomic regions. **(D)** Lollipop chart displaying the proportion of constitutive and dynamic EEs in GBM-specific active regions and LGG-specific active regions, Chi-square test p<0.001***.

**Supplementary Figure 2. UCSC Genome Browser (GRCh37/hg19) 2Mb view around EE-015 and EE-021, including the difference between GBM and LGG ATAC-seq chromatin accessibility peaks. (A)** EE-015 (in yellow) encompasses an area with 26 genes, including those associated with GBM progression, such as *VEGFA*^23^, *RUNX2*^24^, and *CDC5L*^25^. **(B)** EE-021 (in pink) contains 3 GMB-related genes within 2Mb around: PRMT8^44^, FOXM1^45^ and TEAD4^46^. All GBM-related genes around 2Mb of each EE are represented in red. The GeneHancer track indicated EE candidate-gene interactions. High-confidence interactions are shown with solid lines, while low-confidence and reverse-direction interactions are represented by dashed lines.

**Supplementary Figure 3. Comparison of top five predicted TF binding affinities between wild-type and mutated sequence of dynamic EE candidates, including binding motifs.** We considered 15 bp of the sequence being the alteration (red) the central position. **(A, left)** chr6:44635140A>G increased the predicted binding affinity for HSF1, a TF linked to gliomagenesis^27,28^, and also could create a binding site for E2F1, which has also been implicated in tumorigenesis of GBM and other cancers^29–31^. **(A, right)** chr6:44635202T>A, in EE-015 created stronger predicted binding sites for TFs like HOXB13, which promotes proliferation, migration, and GBM invasion^32^, CDX2, linked to GBM malignant behavior^33,34^; and GATA2, involved in GBM progression^35,36^. **(B, left)** chr7:105984983A>T in EE-019 modulated the affinity of forkhead box proteins, increasing slightly the predicted affinity of FOXP1, which has also been linked to GBM progression^37,38^, and FOXA2, a pioneer TF that maintains an open nucleosome arrangement at the EE^39^. **(B, right)** chr7:105985025G>A of EE-019 showed an increased predicted binding affinity of STAT3, associated with GBM progression and the immune microenvironment^37,40^, and THAP1, a master regulator of endothelial cell proliferation^47^. **(C)** chr12:3227012C>G of EE-021 notably increased the estimated affinity of ZFP57, a TF whose altered expression promotes changes in DNA methylation occurring not only before the GBM onset but also as it progresses to high-grade malignancy^48^, and CTCFL, a nuclear and RNA polymerase binding protein whose increased expression is associated with worse overall survival of patients with gliomas^49^.

**Supplementary Figure 4. Corroboration of alteration presence in EE candidates on the tumor FFPE GBM tissues from HUSE-GBM cohort. (A)** Extended heatmap displaying sequencing status (WT, altered by SNV or non-sequenced) of all tissues from the HUSE-GBM cohort in each non-coding region of interest. **(B)** Barplot of the percentage of GBM tissues with SNVs in each non-coding region of interest. The number on the bars is the GBM tissues successfully sequenced. **(C)** Barplot of the number of GBM tissues with the number of altered EE. **(D)** Barplot of the percentage of GBM tissues according to their agreement status in each genomic region studied.

**Supplementary Figure 5. Comparison between peritumoral and tumor area in GBM tissues from HUSE-GBM cohort.** Electropherogram comparison between peritumoral (left) and tumoral (right) of the **(A)** chr6:44635202T>A region (GBM-EE-015) in GBM-034 tissue; chr7:105985025G>A region (GBM-EE-019) in **(B)** GBM-047 and **(C)** GBM-002; chr12:3227012C>G region (GBM-EE-021) in **(D)** GBM-051 and **(E)** GBM-036.

**SUPPLEMENTARY TABLES**

**Supplementary Table S1. Clinical parameters of TCGA-GBM cohort (GBM n=9 and LGG n=12).**

**Supplementary Table S2. Clinical parameters of SweGBM-1 cohort (n=39).**

**Supplementary Table S3. List of the 30 dynamic EEs affected by non-coding somatic SNVs in GBM according to WGS from SweGBM-1 database.** The table includes de EE coordinates (GrcH37/hg19), the SNV position and its genomic change, and the number of GBM patients from the SweGBM-1 cohort with this SNV.

**Supplementary Table S4. Clinical parameters of HUSE-GBM cohort (n=38).**

**Supplementary Table S5. Sequenced regions of interest and identified non-coding mutations in the HUSE-GBM cohort**. A total of 230 sequences were amplified and mapped to the 5 regions of interest.

**Supplementary Table S6. Primers used in this study in genomic DNA from FFPE GBM samples, their amplicon size, and their annealing temperatures.**

**Supplementary Table S7. Sanger sequencing primers.**

**ADDITIONAL REFERENCES**

1. Corces MR, Granja JM, Shams S, et al. The chromatin accessibility landscape of primary human cancers. *Science*. 2018;362(6413). doi:10.1126/science.aav1898

2. Karolchik D, Baertsch R, Diekhans M, et al. The UCSC Genome Browser Database. *Nucleic Acids Res*. 2003;31(1):51-54. doi:10.1093/nar/gkg129

3. Sakthikumar S, Roy A, Haseeb L, et al. Whole-genome sequencing of glioblastoma reveals enrichment of non-coding constraint mutations in known and novel genes. *Genome Biol*. 2020;21(1):127. doi:10.1186/s13059-020-02035-x

4. Lizio M, Harshbarger J, Shimoji H, et al. Gateways to the FANTOM5 promoter level mammalian expression atlas. *Genome Biol*. 2015;16(1):22. doi:10.1186/s13059-014-0560-6

5. Fornes O, Castro-Mondragon JA, Khan A, et al. JASPAR 2020: update of the open-access database of transcription factor binding profiles. *Nucleic Acids Res*. 2020;48(D1):D87-D92. doi:10.1093/nar/gkz1001

6. Yu G, Wang L-G, He Q-Y. ChIPseeker: an R/Bioconductor package for ChIP peak annotation, comparison and visualization. *Bioinformatics*. 2015;31(14):2382-2383. doi:10.1093/bioinformatics/btv145

7. Sherry ST, Ward MH, Kholodov M, et al. dbSNP: the NCBI database of genetic variation. *Nucleic Acids Res*. 2001;29(1):308-311. doi:10.1093/nar/29.1.308

8. Quinlan AR, Hall IM. BEDTools: a flexible suite of utilities for comparing genomic features. *Bioinformatics*. 2010;26(6):841-842. doi:10.1093/bioinformatics/btq033

9. Galaxy Community. The Galaxy platform for accessible, reproducible and collaborative biomedical analyses: 2022 update. *Nucleic Acids Res*. 2022:W345-W351. doi:10.1093/nar/gkac247

10. Fishilevich S, Nudel R, Rappaport N, et al. GeneHancer: genome-wide integration of enhancers and target genes in GeneCards. *Database (Oxford)*. 2017;2017. doi:10.1093/database/bax028

11. Wickham H. Ggplot2: Elegant graphics for data analysis (2nd ed.). *Springer Int Publ*. 2016.

12. Conway JR, Lex A, Gehlenborg N. UpSetR: an R package for the visualization of intersecting sets and their properties. *Bioinformatics*. 2017;33(18):2938-2940. doi:10.1093/bioinformatics/btx364

13. Wong KM, Hudson TJ, McPherson JD. Unraveling the genetics of cancer: genome sequencing and beyond. *Annu Rev Genomics Hum Genet*. 2011;12:407-430. doi:10.1146/annurev-genom-082509-141532

14. Kikutake C, Yoshihara M, Suyama M. Pan-cancer analysis of non-coding recurrent mutations and their possible involvement in cancer pathogenesis. *NAR cancer*. 2021;3(1):zcab008. doi:10.1093/narcan/zcab008

15. Khurana E, Fu Y, Chakravarty D, Demichelis F, Rubin MA, Gerstein M. Role of non-coding sequence variants in cancer. *Nat Rev Genet*. 2016;17(2):93-108. doi:10.1038/nrg.2015.17

16. Zhang X, Meyerson M. Illuminating the noncoding genome in cancer. *Nat cancer*. 2020;1(9):864-872. doi:10.1038/s43018-020-00114-3

17. Pinoli P, Stamoulakatou E, Nguyen A-P, Rodríguez Martínez M, Ceri S. Pan-cancer analysis of somatic mutations and epigenetic alterations in insulated neighbourhood boundaries. *PLoS One*. 2020;15(1):e0227180. doi:10.1371/journal.pone.0227180

18. Osman N, Shawky A-E-M, Brylinski M. Exploring the effects of genetic variation on gene regulation in cancer in the context of 3D genome structure. *BMC genomic data*. 2022;23(1):13. doi:10.1186/s12863-021-01021-x

19. Iñiguez-Muñoz S, Llinàs-Arias P, Ensenyat-Mendez M, et al. Hidden secrets of the cancer genome: unlocking the impact of non-coding mutations in gene regulatory elements. *Cell Mol Life Sci*. 2024;81(1):274. doi:10.1007/s00018-024-05314-z

20. Vinagre J, Almeida A, Pópulo H, et al. Frequency of TERT promoter mutations in human cancers. *Nat Commun*. 2013;4:2185. doi:10.1038/ncomms3185

21. Spiegl-Kreinecker S, Lötsch D, Ghanim B, et al. Prognostic quality of activating TERT promoter mutations in glioblastoma: interaction with the rs2853669 polymorphism and patient age at diagnosis. *Neuro Oncol*. 2015;17(9):1231-1240. doi:10.1093/neuonc/nov010

22. Bell RJA, Rube HT, Kreig A, et al. Cancer. The transcription factor GABP selectively binds and activates the mutant TERT promoter in cancer. *Science*. 2015;348(6238):1036-1039. doi:10.1126/science.aab0015

23. Tian Y, Gao X, Yang X, Chen S, Ren Y. VEGFA contributes to tumor property of glioblastoma cells by promoting differentiation of myeloid-derived suppressor cells. *BMC Cancer*. 2024;24(1):1040. doi:10.1186/s12885-024-12803-8

24. Vladimirova V, Waha A, Lückerath K, Pesheva P, Probstmeier R. Runx2 is expressed in human glioma cells and mediates the expression of galectin-3. *J Neurosci Res*. 2008;86(11):2450-2461. doi:10.1002/jnr.21686

25. Chen W, Zhang L, Wang Y, et al. Expression of CDC5L is associated with tumor progression in gliomas. *Tumour Biol J Int Soc Oncodevelopmental Biol Med*. 2016;37(3):4093-4103. doi:10.1007/s13277-015-4088-5

26. Pridham KJ, Varghese RT, Sheng Z. The Role of Class IA Phosphatidylinositol-4,5-Bisphosphate 3-Kinase Catalytic Subunits in Glioblastoma. *Front Oncol*. 2017;7:312. doi:10.3389/fonc.2017.00312

27. Dai B, Gong A, Jing Z, et al. Forkhead box M1 is regulated by heat shock factor 1 and promotes glioma cells survival under heat shock stress. *J Biol Chem*. 2013;288(3):1634-1642. doi:10.1074/jbc.M112.379362

28. Yuan B, Xu Y, Zheng S. PLOD1 acts as a tumor promoter in glioma via activation of the HSF1 signaling pathway. *Mol Cell Biochem*. 2022;477(2):549-557. doi:10.1007/s11010-021-04289-w

29. Wang X, Wang H, Xu J, Hou X, Zhan H, Zhen Y. Double-targeting CDCA8 and E2F1 inhibits the growth and migration of malignant glioma. *Cell Death Dis*. 2021;12(2):146. doi:10.1038/s41419-021-03405-4

30. Donaires FS, Godoy PRD V, Leandro GS, Puthier D, Sakamoto-Hojo ET. E2F transcription factors associated with up-regulated genes in glioblastoma. *Cancer Biomark*. 2017;18(2):199-208. doi:10.3233/CBM-161628

31. Yoon J, Grinchuk O V, Tirado-Magallanes R, et al. E2F and STAT3 provide transcriptional synergy for histone variant H2AZ activation to sustain glioblastoma chromatin accessibility and tumorigenicity. *Cell Death Differ*. 2022;29(7):1379-1394. doi:10.1038/s41418-021-00926-5

32. Wang X, Sun Y, Xu T, et al. HOXB13 promotes proliferation, migration, and invasion of glioblastoma through transcriptional upregulation of lncRNA HOXC-AS3. *J Cell Biochem*. 2019;120(9):15527-15537. doi:10.1002/jcb.28819

33. Zhang F, Ruan X, Ma J, et al. DGCR8/ZFAT-AS1 Promotes CDX2 Transcription in a PRC2 Complex-Dependent Manner to Facilitate the Malignant Biological Behavior of Glioma Cells. *Mol Ther*. 2020;28(2):613-630. doi:10.1016/j.ymthe.2019.11.015

34. Li Y, Shao T, Jiang C, et al. Construction and analysis of dynamic transcription factor regulatory networks in the progression of glioma. *Sci Rep*. 2015;5:15953. doi:10.1038/srep15953

35. Wang Z, Yuan H, Sun C, et al. GATA2 promotes glioma progression through EGFR/ERK/Elk-1 pathway. *Med Oncol*. 2015;32(4):87. doi:10.1007/s12032-015-0522-1

36. Fu Y, Liu CJ, Kobayashi DK, et al. GATA2 Regulates Constitutive PD-L1 and PD-L2 Expression in Brain Tumors. *Sci Rep*. 2020;10(1):9027. doi:10.1038/s41598-020-65915-z

37. Sun X, Wang J, Huang M, et al. STAT3 promotes tumour progression in glioma by inducing FOXP1 transcription. *J Cell Mol Med*. 2018;22(11):5629-5638. doi:10.1111/jcmm.13837

38. Xue L, Yue S, Zhang J. FOXP1 has a low expression in human gliomas and its overexpression inhibits proliferation, invasion and migration of human glioma U251 cells. *Mol Med Rep*. 2014;10(1):467-472. doi:10.3892/mmr.2014.2197

39. Iwafuchi-Doi M, Donahue G, Kakumanu A, et al. The Pioneer Transcription Factor FoxA Maintains an Accessible Nucleosome Configuration at Enhancers for Tissue-Specific Gene Activation. *Mol Cell*. 2016;62(1):79-91. doi:10.1016/j.molcel.2016.03.001

40. Piperi C, Papavassiliou KA, Papavassiliou AG. Pivotal Role of STAT3 in Shaping Glioblastoma Immune Microenvironment. *Cells*. 2019;8(11). doi:10.3390/cells8111398

41. Yan T, Hu P, Lv S, et al. ZNF384 transcriptionally activated MGST1 to confer TMZ resistance of glioma cells by negatively regulating ferroptosis. *Cancer Chemother Pharmacol*. 2024;94(3):323-336. doi:10.1007/s00280-024-04681-5

42. Yang J, Yang S, Cai J, et al. A Transcription Factor ZNF384, Regulated by LINC00265, Activates the Expression of IFI30 to Stimulate Malignant Progression in Glioma. *ACS Chem Neurosci*. 2024;15(2):290-299. doi:10.1021/acschemneuro.3c00562

43. Bhat KPL, Salazar KL, Balasubramaniyan V, et al. The transcriptional coactivator TAZ regulates mesenchymal differentiation in malignant glioma. *Genes Dev*. 2011;25(24):2594-2609. doi:10.1101/gad.176800.111

44. Simandi Z, Czipa E, Horvath A, et al. PRMT1 and PRMT8 regulate retinoic acid-dependent neuronal differentiation with implications to neuropathology. *Stem Cells*. 2015;33(3):726-741. doi:10.1002/stem.1894

45. Wang Z, Zhang S, Siu TL, Huang S. Glioblastoma multiforme formation and EMT: role of FoxM1 transcription factor. *Curr Pharm Des*. 2015;21(10):1268-1271. doi:10.2174/1381612821666141211115949

46. Xu A, Wang X, Zeng Y, et al. Overexpression of TEAD4 correlates with poor prognosis of glioma and promotes cell invasion. *Int J Clin Exp Pathol*. 2018;11(10):4827-4835.

47. Cayrol C, Lacroix C, Mathe C, et al. The THAP-zinc finger protein THAP1 regulates endothelial cell proliferation through modulation of pRB/E2F cell-cycle target genes. *Blood*. 2007;109(2):584-594. doi:10.1182/blood-2006-03-012013

48. Cirillo A, Di Salle A, Petillo O, et al. High grade glioblastoma is associated with aberrant expression of ZFP57, a protein involved in gene imprinting, and of CPT1A and CPT1C that regulate fatty acid metabolism. *Cancer Biol Ther*. 2014;15(6):735-741. doi:10.4161/cbt.28408

49. Li X, Ning L, Zhang Q, et al. Expression profile of ACTL8, CTCFL, OIP5 and XAGE3 in glioma and their prognostic significance: a retrospective clinical study. *Am J Transl Res*. 2020;12(12):7782-7796.
