# Supplementary figures and images for: Non-coding somatic single-nucleotide variations affecting glioblastoma-specific enhancer elements regulate tumor-promoting gene networks
Source: Genes Dis. 2025 Jul 5;13(1):101762. doi: 10.1016/j.gendis.2025.101762 (PMC12466127; doi:10.1016/j.gendis.2025.101762)

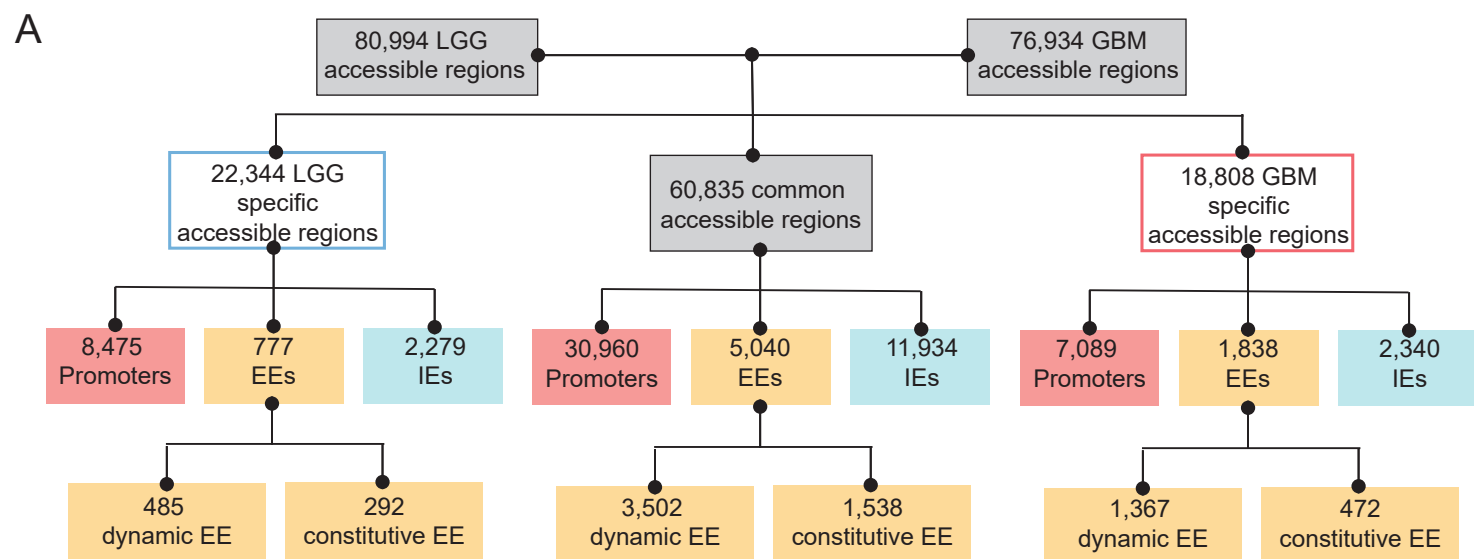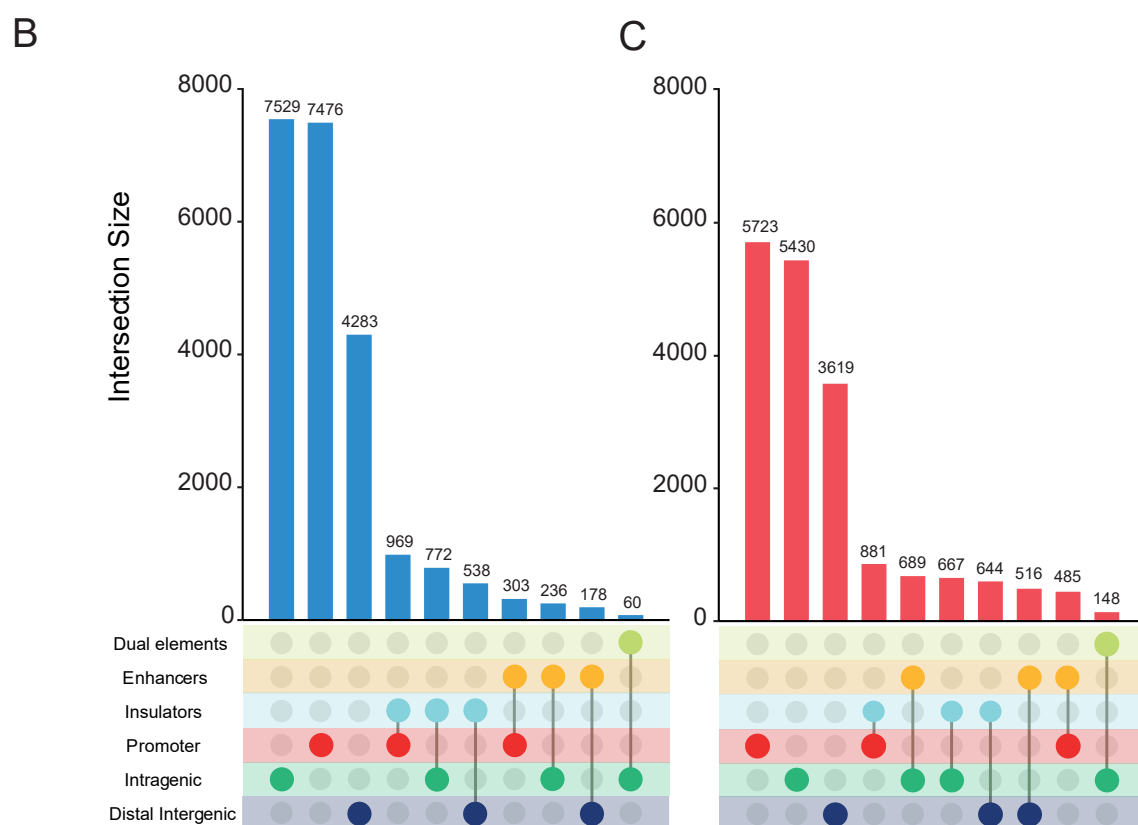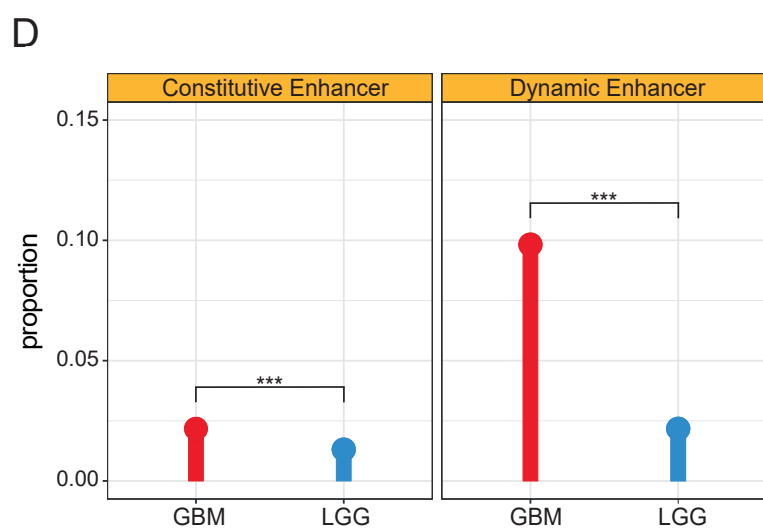

Supplementary figure 1.

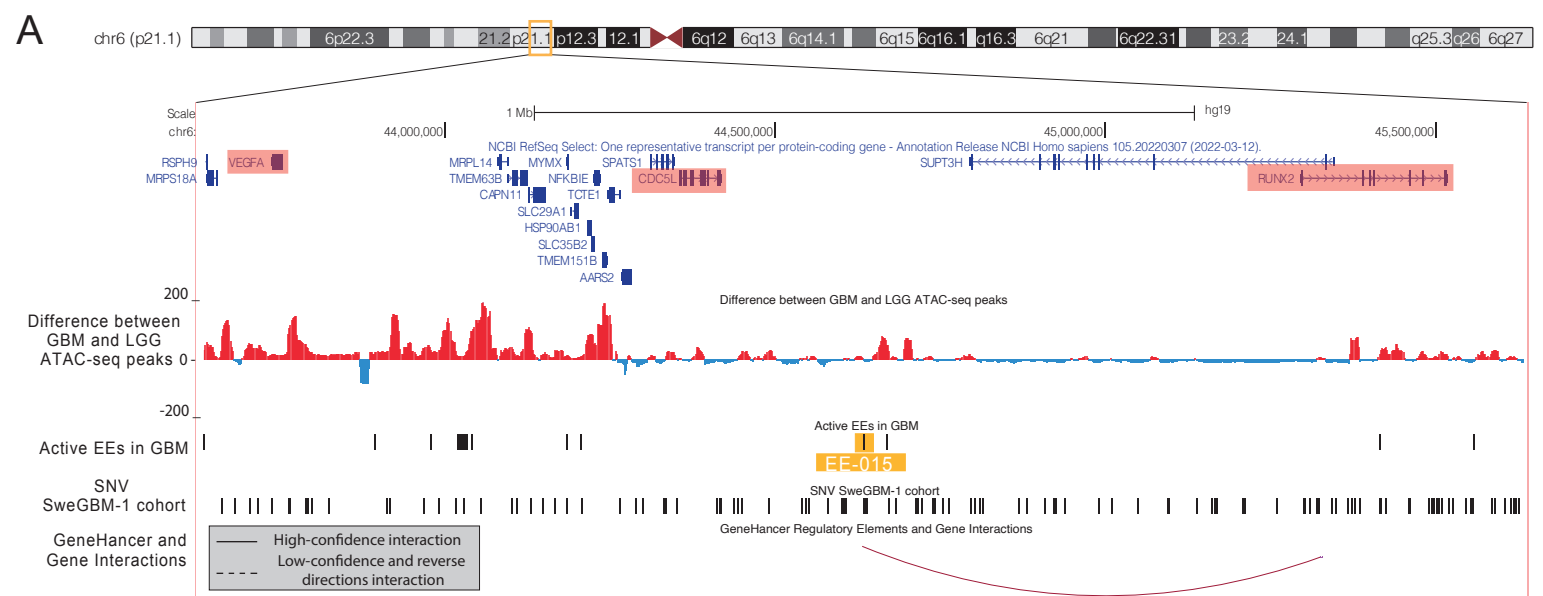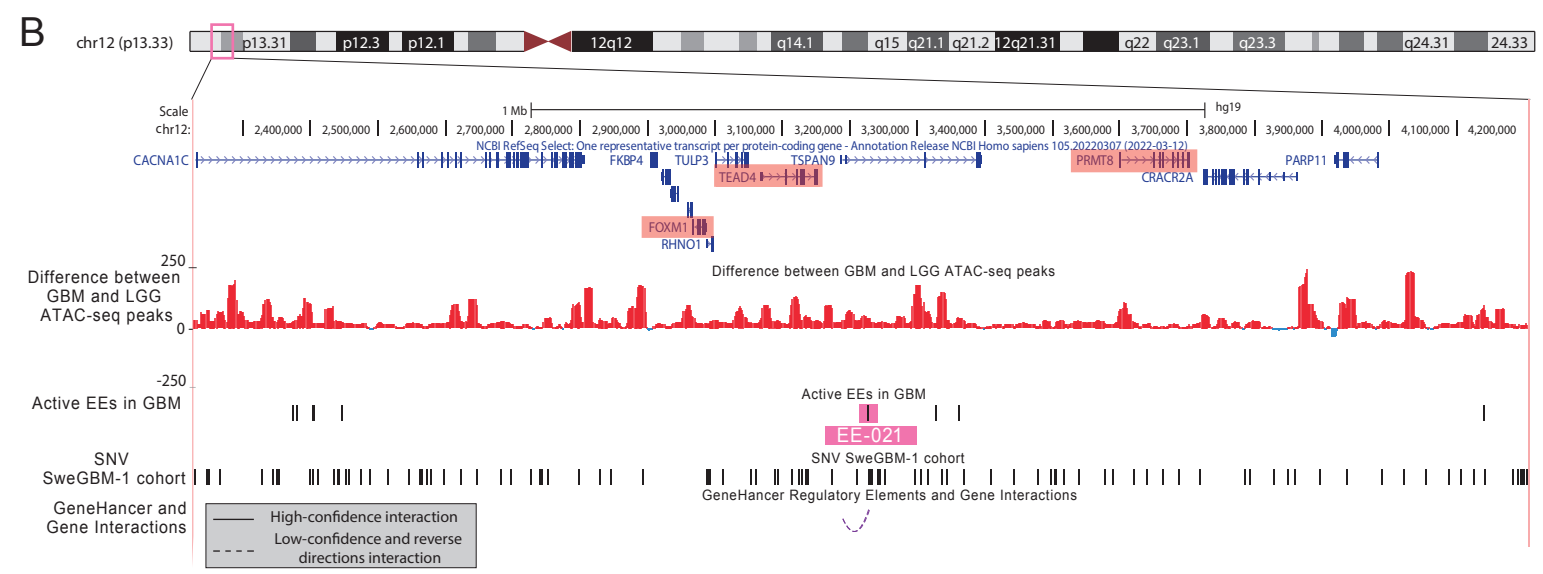

Supplementary Figure 2.

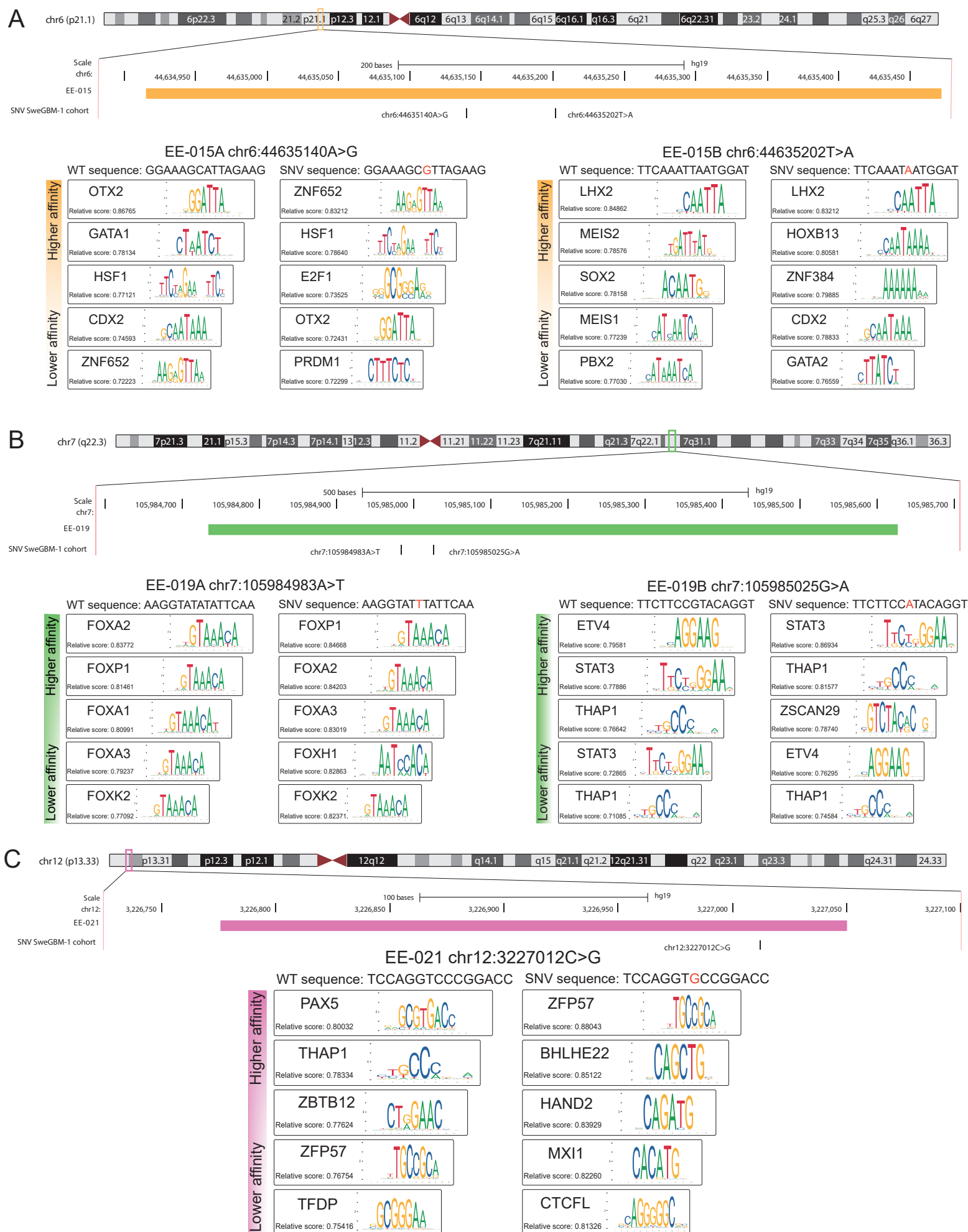

Supplementary Figure 3.

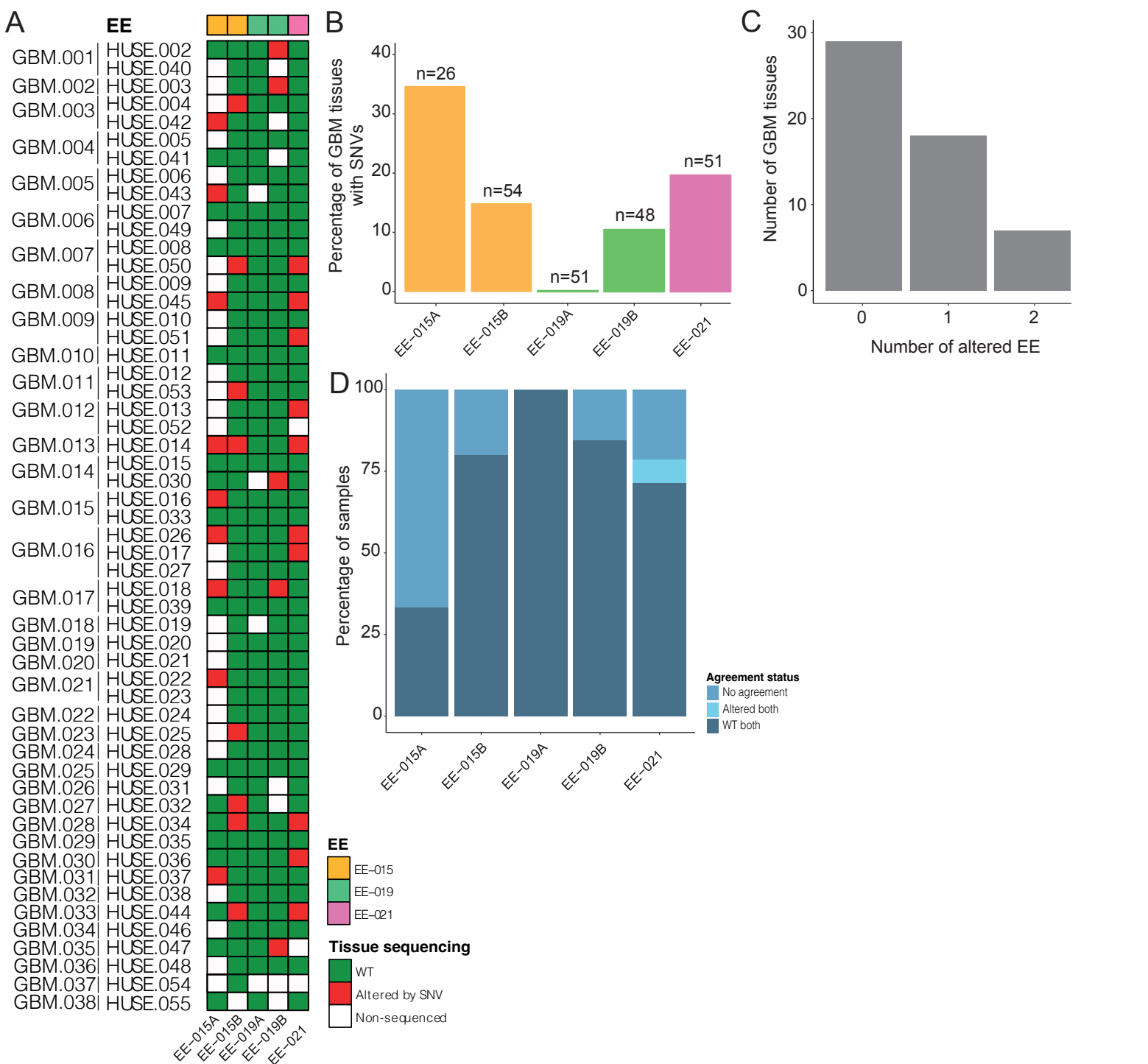

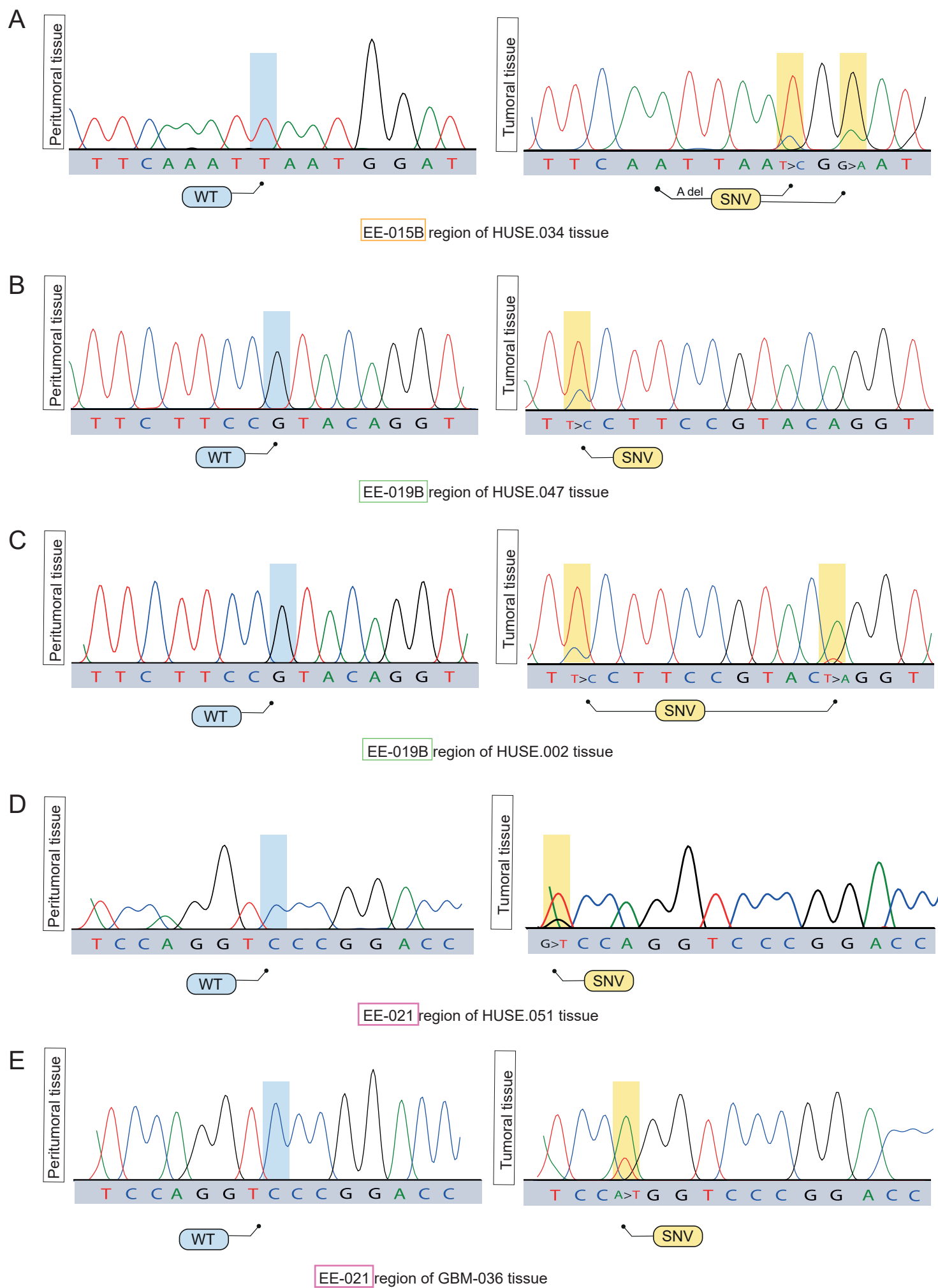

Supplementary Figure 5.

Supplement: Multimedia component 3 [file mmc3.pdf]
